# ACmixSAR-YOLO11: A SAR Ship Detection Model Based on YOLO11

**DOI:** 10.3390/s26216749

**Published:** 2026-10-25

**Authors:** Jiali Li, Baoxiong Xu, Chuanjin Zu, Hao He

**Affiliations:** Ocean College, Jiangsu University of Science and Technology, Zhenjiang 212100, China; 232241803304@stu.just.edu.cn (J.L.);

**Keywords:** SAR ship detection, YOLO11, hybrid attention, noise suppression, multi-scale detection

## Abstract

Hybrid deep learning frameworks integrating convolutional neural networks (CNNs) and Transformer-based attention mechanisms have recently achieved promising results in synthetic aperture radar (SAR) ship detection. However, they still suffer from inherent limitations, including missed detections of small-scale ships under large variations in target scales, multiplicative speckle noise, and complex sea clutter interference. To address these issues, this paper proposes ACmixSAR-YOLO11, a SAR ship detection framework built upon YOLO11. The proposed model incorporates three complementary improvements to enhance feature representation and multi-scale detection capability. First, an ACmixSAR module is designed by integrating convolutional operations with self-attention mechanisms. This design strengthens feature discriminability. Second, a SARNoiseGate gating mechanism is embedded into the ACmixSAR module, which is designed to suppress low signal-to-noise ratio (SNR) feature responses while preserving discriminative ship features. Third, we develop a scale-weighted complete intersection over union (SW-CIoU) loss function, which assigns adaptive regression weights based on target sizes to rebalance the optimization across small, medium, and large ships. All improvements below are reported as relative percentage changes. Experimental results on the SSDD dataset demonstrate that the proposed ACmixSAR-YOLO11 outperforms the original YOLO11 Baseline, achieving a 1.50% increase in precision, a 0.15% growth in recall, a 1.47% enhancement in mAP@0.5, and a 4.26% promotion in mAP@0.5:0.95. Significance testing was applied to mAP@0.5 only (paired one-sided *t*-test, *p* = 0.0058 < 0.01); the other three metrics are reported as three-seed means and spreads. As supplementary evidence, experiments on HRSID show improved recall, mAP@0.5 and mAP@0.5:0.95 under conventional patch-level splitting at the cost of slightly reduced precision. However, this patch-level performance gain is partially inflated by scene overlap between training and test patches. Further validation on a strictly leakage-free scene-level split of HRSID shows that the overall mAP@0.5 gain diminishes to within random seed fluctuation, while improvements in mAP@0.5:0.95 and precision remain observable. These results demonstrate that the proposed framework effectively enhances detection accuracy with a moderate parameter increase (9.69 M → 11.75 M) while maintaining real-time inference.

## 1. Introduction

Synthetic aperture radar (SAR) has become an important remote sensing technology for maritime surveillance owing to its capability to provide all-weather and all-day imaging regardless of illumination and weather conditions. Since it has high spatial resolution and strong penetration capability, SAR has been widely applied to maritime traffic monitoring, fishery management, maritime law enforcement, marine rights protection, and maritime search and rescue [1]. Thus, accurate and fast ship detection from SAR imagery has become an important research topic in ocean remote sensing.

Traditional SAR ship detection methods mainly rely on manually designed statistical rules and hand-extracted features, including constant false alarm rate (CFAR) detection, texture feature extraction, and threshold segmentation techniques [2]. Although these approaches show satisfactory performance in relatively simple sea scenes, they generally suffer from poor robustness in complex environments because handcrafted features cannot effectively represent diverse target characteristics. As a result, they often result in high false alarm rates, severe missed detections, and poor generalization ability in the presence of dense ship distributions, coastal clutter, and large-scale variations. In recent years, with the rapid advancement of deep learning technology, CNN-based methods have become the mainstream for SAR ship detection. According to detection pipelines, these methods can be split into two types: dual-stage and single-stage detectors. Dual-stage detection methods include two core phases: region proposal generation and bounding box prediction. They have heavy computational cost, slow inference speed, and high model complexity, which makes lightweight deployment difficult [3]. One-stage detection algorithms typified by the YOLO series are currently one of the mainstream object detection frameworks and have been widely used due to their outstanding inference speed [4]. These algorithms were originally designed for optical images. When transferred to the SAR ship detection task, they achieve remarkably better ship detection performance than traditional handcrafted feature algorithms. However, affected by SAR speckle noise and complex near-shore backgrounds, these CNN-based detectors still yield unsatisfactory results in many cases [5].

To solve this problem, early studies optimized CNN-based detectors mainly from three aspects: data processing, basic network structure, and bounding box regression loss functions [6,7,8,9,10,11]. Reference [6] proposed a CNN-based method for high-resolution SAR aircraft detection. It first located candidate targets roughly and then achieved accurate detection via CNN. Four SAR data augmentation methods were also proposed to relieve overfitting caused by insufficient SAR samples. Built upon the Single Shot MultiBox Detector (SSD), reference [7] combined transfer learning and sample expansion to improve SAR target detection performance. Reference [8] presented a new CNN-based SAR ship detection method, which improves detection accuracy and inference speed by optimizing multi-scale feature aggregation, pre-trained model transfer and loss functions. Reference [9] integrated traditional machine learning and convolutional networks. The method first uses a support vector machine to roughly extract candidate ship regions of interest. Then it adopts an optimized Faster R-CNN to achieve precise detection. These two steps effectively improve SAR ship detection performance. These works demonstrate the effectiveness of deep learning in SAR ship detection. Reference [10] introduced the Feature Pyramid Network (FPN) into a lightweight SAR ship detection model. It optimizes multi-scale feature fusion, lightweight convolution and adaptive feature weighting to enhance multi-scale detection ability. However, most anchor-based methods in [7,8,9] divide anchor boxes into positive and negative samples by fixed manual IoU thresholds. This strategy ignores the actual overlapping features and cannot adaptively adjust weights for targets of different sizes. Thus, it achieves poor detection results for densely distributed near-shore ships [11]. In addition, the FPN adopted in [10] only adopts a top–down single-way fusion structure without bottom–up feature transmission. As a result, even with these improvements, pure CNN-based methods still find it hard to capture global context and fuse multi-scale features well. This makes it difficult to separate ships from strong coastal clutter in complex scenes [12].

To overcome the limited view range of CNNs and better capture long-range dependencies for improved localization, recent studies have introduced attention mechanisms and Transformer modules to suppress complex background disturbances and highlight valid ship features [13]. Reference [14] proposed an entropy-based resampling strategy for imbalanced SAR object detection [15]. It adopts variance-weighted image entropy and category distribution to reduce the negative impacts of image quality differences and class imbalance. Reference [16] developed a Non-Local Channel Attention Network (NLCANet) to model pixel-region correlations and channel dependencies [17], which addresses the problem of target feature attenuation in complex backgrounds. Reference [18] designed a Feature Interaction Network (FINet) to fuse target-level and pixel-level information, improving ship localization accuracy under speckle noise and clutter interference. In addition, reference [19] integrated Efficient Channel Attention (ECA) [20] and Swin Transformer modules into the backbone of EST-YOLO5s [21], which greatly promotes the detection performance of small SAR ship targets. These works show that combining CNNs with attention or Transformer modules is a promising trend for SAR ship detection, as it uses CNNs for local features and Transformers for global relationships. Similar designs that couple dynamic attention with multi-scale information fusion have also proven effective in broader maritime deep learning tasks beyond detection, such as data-driven ship fuel-consumption prediction [22].

Although current approaches have achieved encouraging advances for SAR ship detection, three key problems still remain in complex nearshore scenes: First, most attention mechanisms only work on one dimension, either channel or space. They cannot build two-way connections between spatial and channel features, which weakens feature representation under strong clutter. Also, most attentions are placed in the backbone, while the feature fusion stage, where multi-scale information comes together, receives little attention. Second, modern networks like PANet use two-way fusion, but they simply add or combine features with fixed weights [23]. They do not adaptively adjust the weights according to SAR noise properties. Third, common IoU-based losses use the same gradient weights for all object scales [24]. This makes large ships dominate the learning process, while small ships get much smaller gradients [25]. Because of these problems, existing models cannot capture fine ship features, and small ships in coastal scenes are often missed [26]. To solve these issues, this paper proposes a lightweight SAR ship detection framework named ACmixSAR-YOLO11.

The main contributions are summarized as follows:

1. Focusing on the disturbance caused by ocean clutter interference and weak small ship features in SAR images, this paper embeds the Asymmetric Convolution Mix Attention (ACmix) into the neck feature fusion network of YOLO11. The module works at the neck level to enhance feature representation through adaptive fusion of multi-scale information.
2. To suppress multiplicative speckle noise in SAR images, an improved ACmixSAR hybrid attention module is proposed in this paper. A SARNoiseGate gating unit is introduced into the self-attention branch of the original ACmix to suppress clutter channels that hurt the signal-to-noise ratio. This helps strengthen the features of real ships.
3. To address the challenges of diverse ship sizes and poor convergence of bounding box regression for tiny ships in SAR port scenes, this paper introduces an SW-CIoU into the traditional CIoU framework. It assigns loss weights according to the area ratio of ground-truth ship boxes to the whole image, which balances the optimization weight difference between large and small ships.

The rest of the paper is structured as follows. Section 2 describes the role of the proposed algorithm in SAR ship detection. Section 3 presents the experimental setup, dataset, and evaluation metrics. Section 4 presents experimental results and discussion. Finally, Section 5 concludes this paper.

## 2. Proposed Algorithm

### 2.1. Overall Architecture of ACmixSAR-YOLO11

**Figure 1:**
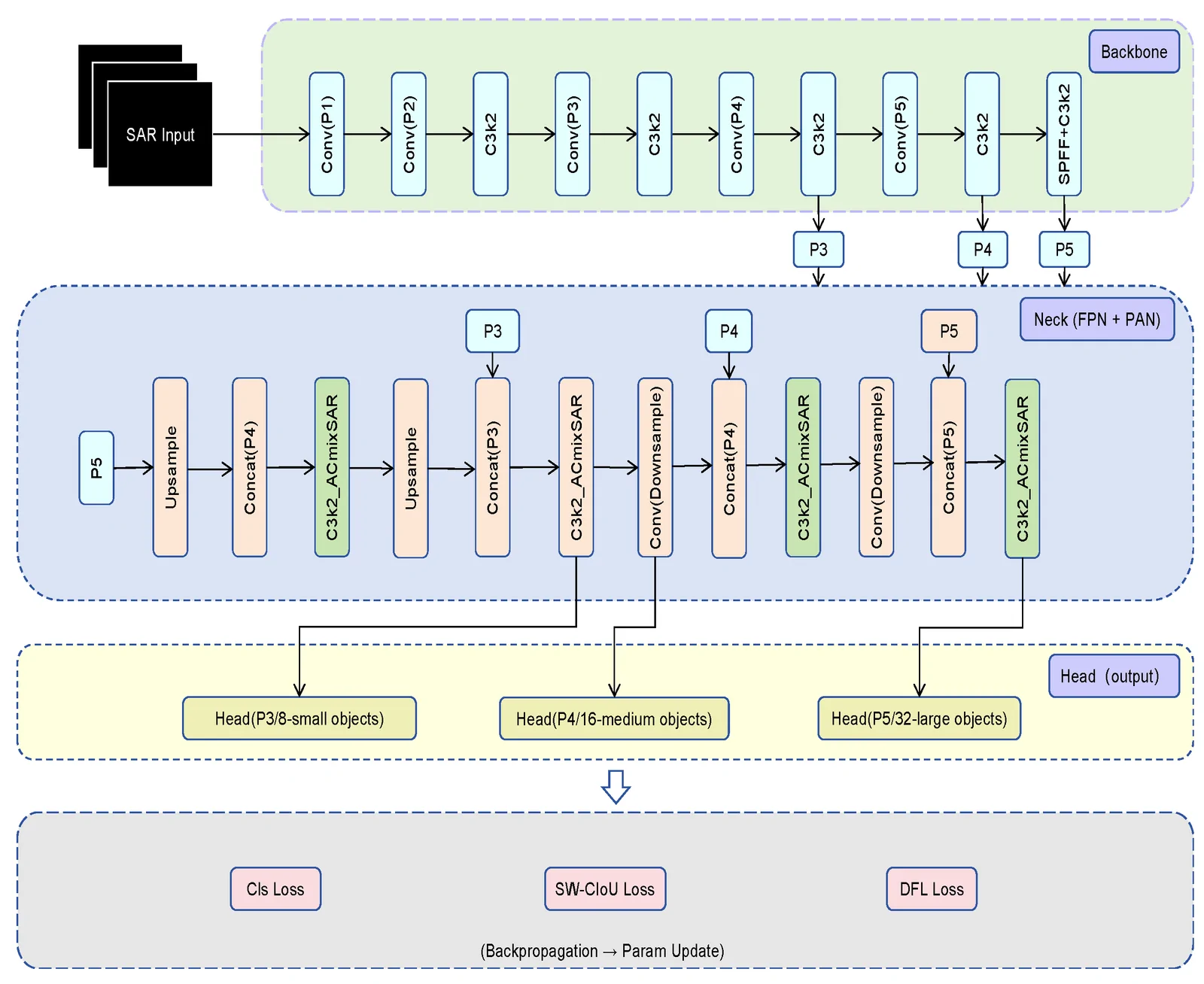
Overall model: ACmixSAR-YOLO11.

SAR ship detection remains challenging because of multiplicative speckle noise, complex coastal background clutter imbalanced multi-scale target distribution, and inconsistent global–local feature characterization. To address these challenges, we propose a SAR ship detection method embedded with the ACmixSAR module based on YOLO11, which is a lightweight SAR ship detection framework. Figure 1 shows the overall framework of the proposed algorithm. The model takes SAR ship images as input and outputs the bounding boxes, category labels, and confidence scores of ships. The framework mainly contains a backbone network, a neck feature fusion network, and a detection head. First, SAR ship images are fed into the backbone, which outputs three feature maps P3, P4, and P5, with spatial resolutions of 1/8, 1/16, and 1/32 of the input image, respectively. Second, these three-scale feature maps are passed to the neck feature fusion network. The ACmixSAR module embedded in each C3k2_ACmixSAR block of the neck initially extracts local geometric features via the convolution branch while simultaneously modeling global features via the self-attention branch. The module then applies SARNoiseGate to adaptively modulate the value vectors of the self-attention branch, thereby purifying the global features. Finally, it employs dynamic weights and residual connections to adaptively fuse local and global features, which suppresses noise-contaminated channels and strengthens ship target responses. The neck module outputs three optimized feature maps and transmits them to the detection head. This detection branch outputs three feature tensors for different scales. Each tensor contains three kinds of parameters: four parameters represent bounding box offsets, one parameter represents target confidence, and nc parameters represent the number of categories. During training, the SW-CIoU loss function supervises the output by computing the geometric deviation between predicted and ground truth boxes. This mechanism drives gradient backpropagation and optimizes the network parameters.

### 2.2. ACmix Hybrid Attention Module

**Figure 2:**
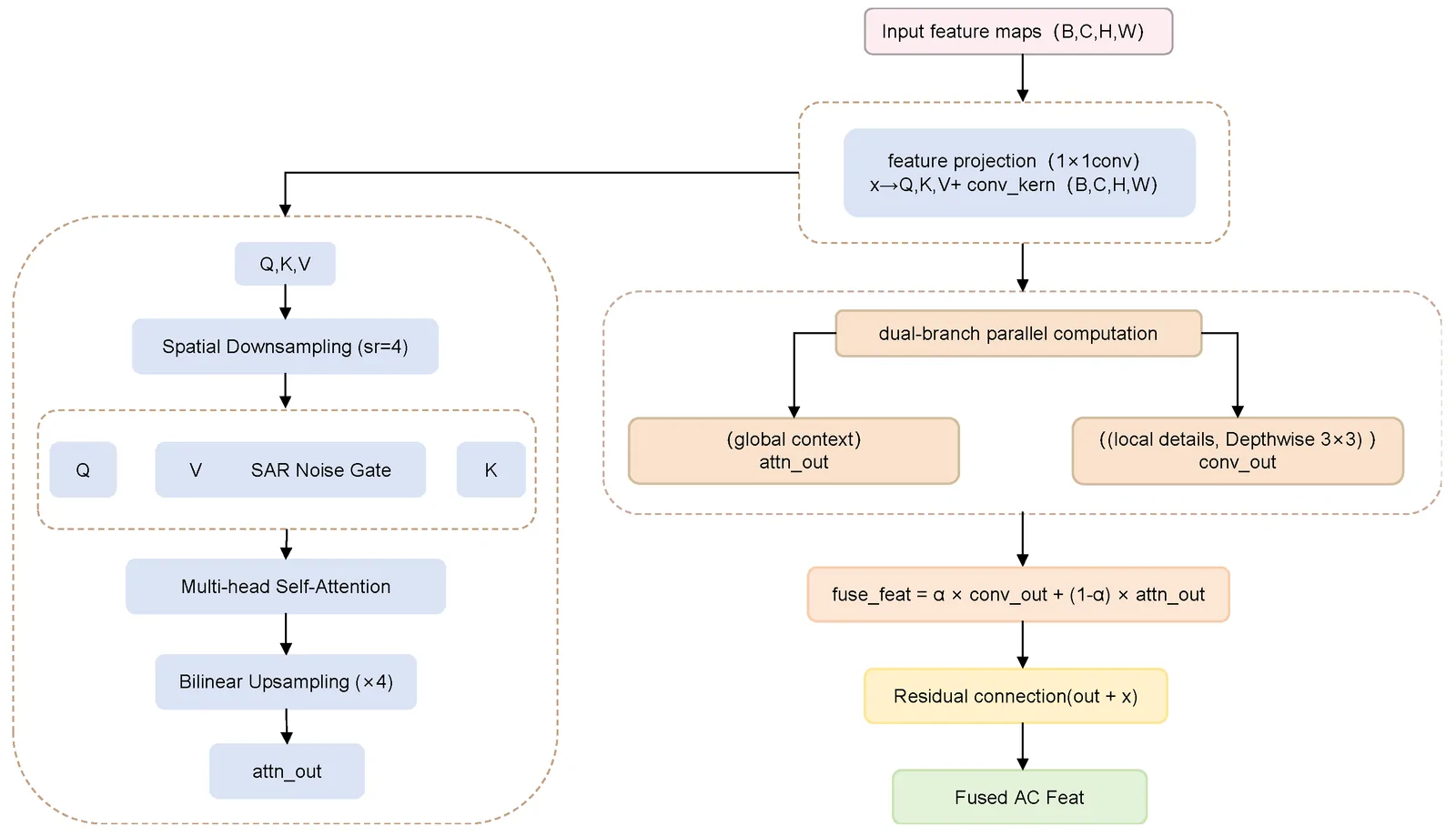
Architecture of the proposed ACmixSAR module.

The proposed ACmixSAR module is developed based on the original ACmix architecture by introducing an SAR-oriented feature enhancement mechanism. ACmix was originally designed as a backbone module for image classification tasks [27]. The core innovation of this work is not the ACmix operator itself but the coupling strategy that embeds a Noise Gate into the attention branch, as well as the neck-only deployment with unchanged channel dimensions. The original ACmix increases channel dimensions in the backbone network. Different from this practice, we apply ACmixSAR to the C3k2 blocks of the detection neck (P3–P5) while keeping the channel dimension unchanged, which avoids the FLOPs overhead introduced by the original channel-expansion design. Most importantly, we embed a SARNoiseGate module inside each ACmix bottleneck to modulate the value vectors of the self-attention branch. Figure 2 shows the overall structure of the proposed ACmixSAR module.

In the core workflow of the ACmixSAR module, it first uses a shared 1 × 1 convolution projection to generate queries (Q), keys (K), values (V), and reconstruction kernels for the convolution branch. The feature extraction process is divided into two parallel paths. The convolution branch uses spatial shifting and reconstruction kernels to extract local geometric features, focusing on capturing the rigid contour information of ship targets, such as edges and bulwarks. The self-attention branch suppresses the interference of speckle noise on global modeling and outputs purified global contextual features. To achieve adaptive fusion of the two branches, we propose a learnable dynamic complementary gate. Compared with the conventional Squeeze-and-Excitation (SE) block, the proposed gating mechanism improves in three aspects: gating mode, residual structure and domain adaptability. The standard SE block enhances valid features via $x_{out}=x⊙s$ with a trivial identity shortcut, which is a domain-agnostic design. By contrast, our design employs two complementary gating mechanisms: a SARNoiseGate that suppresses noisy features through $V_{gated}=V⊙\left(1-g_{n}\right)$ applied to the value tensor of the attention branch and a dynamic fusion gate that adaptively balances the convolution and attention branches through

(1) $$X_{out}=X_{conv}⊙g_{f}+X_{att}⊙\left(1-g_{f}\right)+X,$$

where $g_{f}=\sigma \left(Conv_{1\times 1}\left(GAP\left(X\right)\right)\right)$ is the fusion gate weight computed via global average pooling and a learnable 1×1 convolution, $X_{conv}$ is the convolution branch output, *X_att_* is the attention branch output (with noise-gated values), and X is the residual connection. The gate weights adapt to the statistical properties of SAR speckle noise through end-to-end training on SAR data.

The adaptive fusion coefficients are computed as

(2) $$g_{f}=\sigma \left(Conv_{1\times 1}\left(GAP\left(X\right)\right)\right),$$

where ⊙ denotes channel-wise multiplication, $\sigma \left(\cdot \right)$ denotes the Sigmoid function, and $\mathrm{GAP}\left(\cdot \right)$ denotes global average pooling. In texture-rich sea surface areas, the gate weights automatically increase, placing more emphasis on local convolutional details. In open sea areas with large-scale scenes, the gate weights automatically decrease, placing more emphasis on global contextual dependencies.

### 2.3. SARNoiseGate for Noise-Aware Feature Recalibration

**Figure 3:**
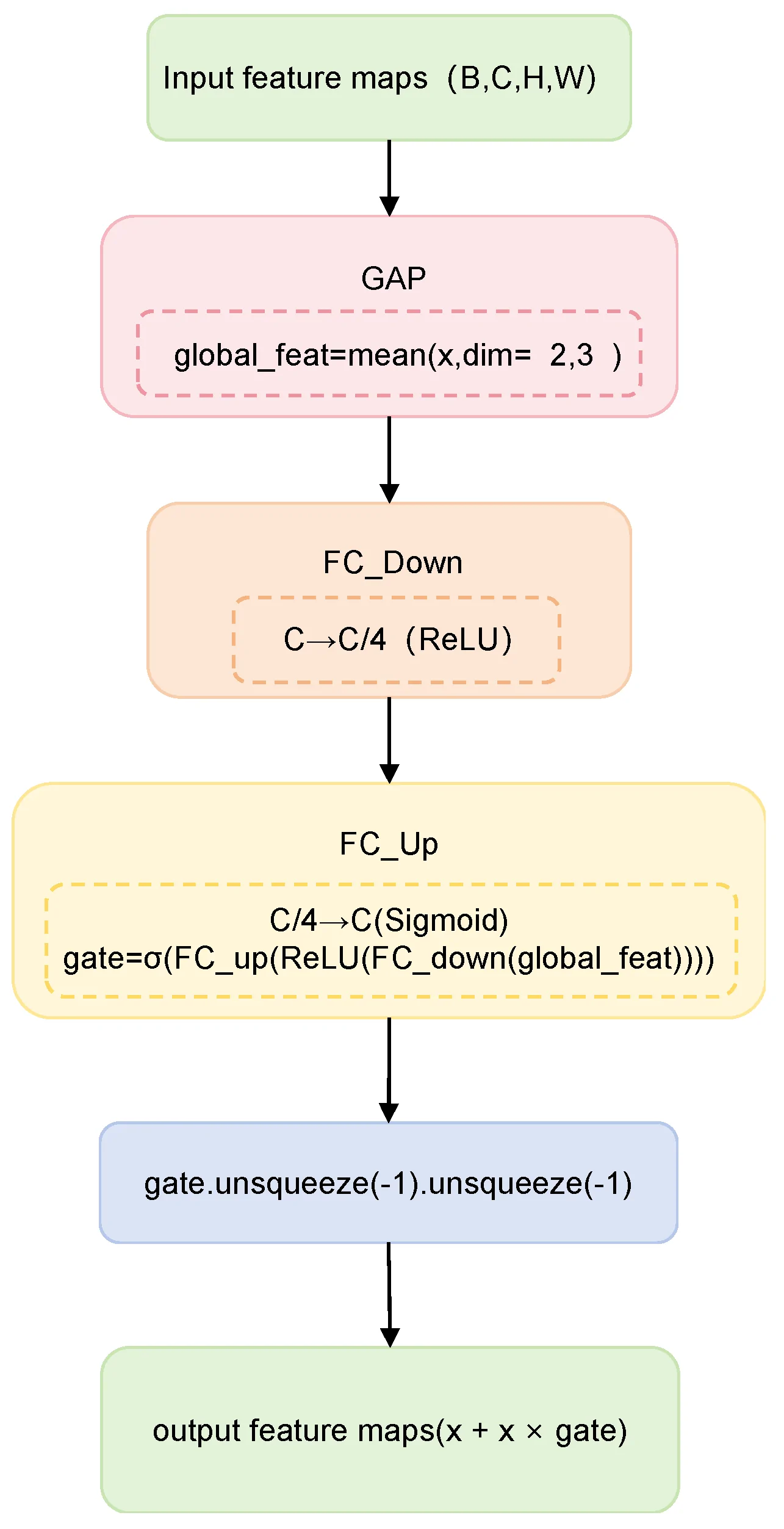
Architecture of the proposed SAR Noise Gate module.

Based on the above design, the original self-attention branch of ACmix suffers from a drawback. Clutter channels produce ineffective high responses. In port scenes, tiny fishing vessels occupy very few pixels. Their weak scattering features are completely masked by these ineffective responses. Traditional SE [28] Channel Attention only provides general feature enhancement. It does not have suppression logic designed for SAR noise. To address this issue, we introduce the SARNoiseGate noise suppression unit, as shown in Figure 3. We embed it into the value vectors of the self-attention branch of ACmix. This forms the ACmixSAR module. This module adaptively suppresses the weights of noisy channels. At the same time, it relatively amplifies the channel features of ship targets in SAR ship detection.

#### 2.3.1. Global Squeeze

The input of this module is a four-dimensional feature tensor $x\in {\mathbb{R}}^{B\times C\times H\times W}$, where B refers to the batch size, C represents the number of feature channels, and H and W denote the height and width of feature maps, respectively. First, to precisely quantify the impact of every single channel on vessel detection, we apply global average pooling to the spatial dimensions of the input feature vectors. This compresses the spatial information of each channel into a single scalar, obtaining the global statistical representation of each channel:

(3) $$g=\frac{1}{H\cdot W}\sum_{h=1}^{H}\sum_{w=1}^{W}\chi_{b,c,h,w},$$

where the value g is the single value used to stand for one whole feature channel, H⋅W means all pixel points on the feature map, and $\chi_{b,c,h,w}$ is the feature value at position $\left(h,w\right)$ of channel c in batch b. This calculation turns all space information of each channel into one simple value, so we can judge how useful each channel is for ship detection.

#### 2.3.2. Channel Squeeze and Excitation

A dimensionality reduction fully connected layer is used to reduce the number of parameters, and ReLU is adopted to introduce nonlinearity:

(4) gmid=ReLUWdowng+bdown,

where $W_{down}\in R^{\left(C/4\right)\times C}$, and such an operation enables the network to focus on distinguishing ship targets from sea clutter in a low-dimensional space. It compresses the channels to C/4, reducing the computational cost.

#### 2.3.3. Channel Restoration and Sigmoid Gating Generation

After dimensionality reduction, to restore the independent gating coefficient of each channel, we expand the dimension size to match the original channel count. Afterwards, the Sigmoid activation limits the output values within $\left[0,1\right]$, yielding the suppression weight of each channel:

(5) $$gate=\sigma \left(W_{up}g_{mid}+b_{up}\right),$$

where $\sigma \left(\cdot \right)$ denotes the Sigmoid activation function, $gate_{b,c}\in \left[0,1\right]$. The learned gate values are inversely related to feature saliency: a value closer to 1 indicates a noise-dominated channel that should be suppressed, while a value closer to 0 indicates a target-relevant channel that should be preserved.

#### 2.3.4. Feature Channel Modulation

Finally, the original features are multiplied channel-wise with the gating weights, completing the noise suppression:

(6) $$x_{denoise}=x⊙\left(1-G\left(gate⊗1_{H\times W}\right)\right),$$

where ⊙ denotes the channel-wise Hadamard product. For channels corresponding to sea clutter, the feature responses are significantly suppressed. For channels with strong ship scattering, the features are fully preserved.

#### 2.3.5. Statistical Validation for SAR Speckle Coupling Analysis

This work introduces a dedicated gating mechanism for SAR imagery. Different from generic SE-like modules, this mechanism is specifically tailored for SAR speckle noise. We conduct three statistical analyses to verify the inherent correlation between the Noise Gate (NG) and SAR speckle features. Table 1 summarizes all quantitative validation results.

These analyses validate two core characteristics of the proposed NG. First, the NG is inherently coupled with SAR speckle statistics. Second, its working mechanism is fundamentally different from conventional SE blocks. Both SSDD and HRSID datasets conform to the Gamma speckle distribution, with KL divergence values below 0.03. This distribution pattern aligns with the standard multiplicative speckle model of SAR images.

To further investigate the coupling between the Noise Gate and SAR speckle noise, we perform a statistical analysis on the test set. For each image we compute a patch-level signal-to-noise ratio $SNR\;=\;sqrt\left(ENL\right)\;=\;sqrt\left(\mu^{2}/\sigma^{2}\right)$ over annotated ship regions, where μ and $\bar{g}$ are the local mean and variance within a 16 × 16 window, and extract the mean gate activation $\bar{g}$ (fusion-gate α) of the NG at each feature depth. Across the four gated feature layers, the Pearson correlation between the SNR and the gate output is layer-dependent in sign (layer-wise r from −0.67 to +0.81, |r| = 0.42–0.81, all *p* < 0.001); we therefore present these results as evidence of a layer-specific association only, without claiming a causal suppression mechanism. On SSDD, the global gate–SNR correlation is trivial and non-significant (r = −0.01, *p* = 0.87).

ENL evaluation further explains the performance discrepancy. SSDD (4.00) and HRSID (4.07) exhibit similar median ENL values, indicating comparable overall speckle intensity. However, SSDD shows a significantly higher ENL variance (34.26) compared to HRSID (2.43). This high variance stems from the diverse scene types in SSDD, which includes open sea, harbor areas, and complex coastlines. Consequently, the NG’s gating behavior is strongly coupled with local SNR on HRSID (|r| up to 0.81), whereas the coupling is weak and non-significant on SSDD, consistent with SSDD’s much higher ENL variance. By contrast, the more uniform speckle noise in HRSID enables the NG to learn stable and effective suppression patterns across the entire dataset.

### 2.4. Small-Scale CIoU Loss for Small-Scale Ship Localization

**Figure 4:**
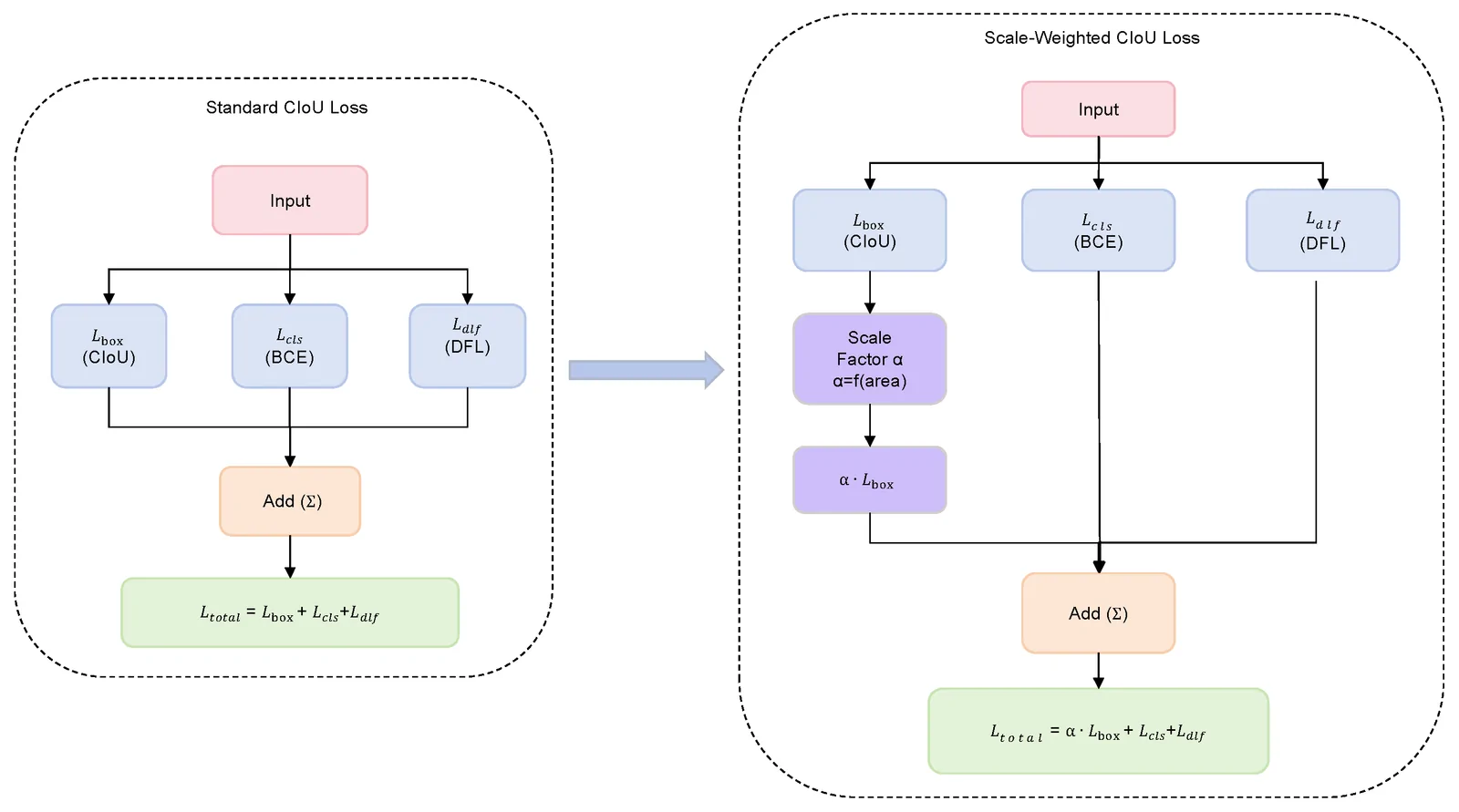
Small-scale CIoU loss model.

The original CIoU loss assigns equal weights to all objects regardless of their scale. In SAR ship detection, however, tiny fishing vessels occupy only a few pixels and contribute much smaller gradient magnitudes than large cargo ships, causing the training signal to be dominated by large targets. This scale imbalance leads to severe missed detections and localization errors for small ships [29]. Therefore, we introduce adaptive scale weighting, as shown in Figure 4.

#### 2.4.1. Relative Area Ratio of Ships

The input images are resized to a uniform size. Let S×S be the reference pixel area. The proposed loss function is formulated as

(7) $$r=\frac{Area\left(B_{gt}\right)}{S\times S},$$

where $Area\left(B_{gt}\right)$ is the pixel area of the ground-truth ship box and r is the ratio of the actual ship area to the reference area. This ratio helps distinguish large, medium, and small ships and assign different loss weights accordingly.

Statistical analysis shows that 79.6% of ship instances in SSDD and 96.9% in HRSID have bounding box area ratios below 1%, indicating a structural deficiency in sensitivity to extremely small-scale targets.

#### 2.4.2. Piecewise Adaptive Weighting Function

To balance the training loss of ships with different sizes, we introduce a scale-adaptive weight factor w defined as

(8) $$w\left\{\begin{array}{l} 1.5\;\;r<0.01\;\;\;\;\mathrm{Small}\;\mathrm{ships}\\ 1.2\;\;0.01\leq r<0.04\;\;\mathrm{Medium}\;\mathrm{ships}\\ 1.0\;\;r\geq 0.04\;\;\;\;\mathrm{Large}\;\mathrm{ships} \end{array}\right.,$$

where r denotes the ratio of the pixel area of each ship bounding box to the total pixel area of the input image. The three thresholds partition the ship population roughly as 80%/15%/5% on SSDD and 97%/2.5%/0.5% on HRSID. The weights (1.5, 1.2, 1.0) are calibrated so that small ships, which account for the overwhelming majority but suffer from vanishing gradients, receive the strongest amplification, while large ships retain the Baseline CIoU weight.

#### 2.4.3. Sensitivity Validation

To verify that the selected weights are optimal and robust, we conduct a 7-point sensitivity sweep on SSDD by varying the small-ship weight $w_{s}\in \left\{1.0,1.2,1.3,1.5,1.7,1.8,2.0\right\}$ while keeping $w_{m}=round\left(0.5+0.5\cdot w_{s}\right)$ and $w_{l}=1.0$ fixed. Table 2 reports the results.

All entries in this table were obtained under a fast-screening protocol (150 epochs, patience 15, batch size 8, lr0 = 5 × 10^−4^, dropout 0.1, augmentation disabled), so that the seven weight configurations are compared under a strictly comparable budget. The W0 entry (all weights = 1.0, i.e., the original CIoU loss) is an in-sweep reference only.

W2 $\left(w_{s}=1.5,w_{m}=1.2,w_{l}=1.0\right)$ achieves the best mAP@0.5 of 0.9804, outperforming the standard CIoU Baseline by +1.50 pp. Therefore, we adopt W2 as the default SW-CIoU configuration. Ablations T0 and T1 isolate the contribution of medium-ship weighting; removing it drops mAP by 0.36–0.89 pp, confirming that moderate amplification of medium ships further improves overall performance. The broad neighborhood around W2 (0.9731–0.9804) confirms that the parameters are robust and non-critical.

#### 2.4.4. Final Scale-Weighted CIoU Loss

Based on the scale-adaptive weight w defined above, we construct the final multi-scale balanced bounding box loss function as

(9) $$L_{scale-CIoU}=\frac{1}{N}\sum_{i=1}^{N}w\left(r_{i}\right)\cdot \left(1-CIoU_{i}\right),$$

where N denotes the overall count of vessel objects within the current batch. After weighting each sample individually, the mean value is taken as the batch bounding box loss.

## 3. Experimental Environment and Datasets

### 3.1. Experimental Environment

All experimental tests in this work were carried out under the Windows 11 OS, equipped with an Intel Core i7-13700KF central processor (Intel Corporation, Santa Clara, CA, USA) and an NVIDIA GeForce RTX 5070 graphics card (NVIDIA Corporation, Santa Clara, California, USA). The PyTorch 2.7.0 deep learning framework built on Python 3.11 with CUDA 12.8 was adopted. Network construction and training were completed via the Ultralytics YOLO library. We adopted the AdamW optimizer to update model parameters. Its initial learning rate was set to 0.0005, weight decay to 0.01, momentum to 0.937, total training epochs to 500, and batch size to 8. The cosine learning rate decay strategy was adopted with 10 warm-up epochs. Automatic mixed precision (AMP) was disabled to ensure consistent numerical behavior across all runs. In order to lower computing costs, all input images were resized to a unified resolution of 416, while the random seed was set to 42 to ensure experimental reproducibility. An early stopping mechanism was also configured, which automatically terminates training when the mAP metric on the validation dataset has no improvement across 50 successive epochs.

For the two-stage training strategy used on HRSID, Stage 1 (NG and ACmix pre-training without SW-CIoU) followed the above protocol for up to 500 epochs. Stage 2 then loaded the Stage-1 best checkpoint, froze all Noise Gate parameters (requires_grad = False), reduced the initial learning rate to 0.0001 for fine-tuning, and ran for up to 150 epochs while enabling the SW-CIoU loss. SSDD results were obtained with the standard joint-training protocol (single stage, 500 epochs, no freezing).

### 3.2. Datasets

To evaluate the detection capability of the proposed method for marine vessel targets, two open-access SAR ship detection datasets, SSDD [30] and HRSID [31], are employed. The detailed information of these datasets is shown in Table 3. SSDD comprises 1160 marine scene images with 2456 ship instances at a 1–15 m resolution. The original HRSID dataset contains 5604 large SAR scenes with 16,951 ship instances at a 0.5–3 m resolution. Without slicing, HRSID scenes exceed 5000 × 10,000 pixels; resizing directly to 416 × 416 would compress typical 5 m fishing boats to sub-pixel size, making them impossible to detect. We therefore apply 800 × 800 overlap slicing and discard empty sub-images, yielding 3642 valid ship-bearing slices that preserve small-target pixel fidelity while removing pure-clutter patches.

Each dataset is split into three parts using a unified 7:2:1 ratio with stratified random sampling (seed=42). If we do not use stratification, rare samples like large ships and port scenes may all end up in the same part. Then the AP numbers would not be stable across runs. If we use different split ratios for the two datasets, we cannot compare them fairly. The split gives 116 test images for SSDD and 365 test images for HRSID, which is enough for stable size-based AP evaluation.

The test set contains 365 patches in total. Among them, 321 test patches (87.9%) are derived from scenes that also appear in the training set. Within the 354 patches with parseable annotations, 310 test patches (87.6%) have spatial overlap with training patches, with a median IoU of 0.14, and nine patches exhibit an IoU > 0.5. To evaluate the impact of this scene overlap, we construct a leakage-free scene-level split for HRSID, as described in Section 4.1.4.

All images are resized to 416 by 416 pixels. This size is chosen carefully. At 320 by 320 pixels, a large portion of HRSID ships become smaller than 2 pixels and cannot be measured by the COCO metric. At 512 by 512 pixels, training with batch size 8 uses more than 8 GB of GPU memory, so most users cannot run it. At 416 by 416, large ships still have enough feature cells, small ships stay visible, and training fits well on common GPUs.

### 3.3. Evaluation Metrics

For comprehensive assessment of the presented ACmixSAR-YOLO network, precision (P), recall (R), mAP@50 and mAP@50:95 are selected as quantitative evaluation indicators. Precision represents the proportion of real ship targets in all detected bounding boxes. Recall indicates the proportion of labeled ship targets successfully detected from the dataset. The relevant formulas are defined as follows:

(10) $$P=\frac{N_{TP}}{N_{TP}+N_{FP}},$$

(11) $$R=\frac{N_{TP}}{N_{TP}+N_{FN}}.$$

True positive refers to the number of correctly detected ship targets. False positive denotes the number of incorrectly detected ship targets. False negative indicates the number of ship targets missed and unrecognized by the model. AP is calculated by integrating the P-R curve, which serves as a core metric for evaluating detection accuracy, in which P stands for precision and R refers to recall. The specific computational formulas are as follows:

(12) $$mAP=AP=\int_{0}^{1}P\left(R\right)dR.$$

To verify the model’s generalization capacity toward vessels with various scales, mAP@50 and mAP@50:95 are calculated in the experiment. mAP@50 represents the average precision calculated at an IoU threshold of 0.5. mAP@50:95 represents the average precision computed at IoU thresholds ranging from 0.5 to 0.95 with a step of 0.05. The IoU threshold refers to the intersection over union metric calculated between predicted bounding boxes and ground truth annotations for object detection tasks. A target is considered successfully detected if the ratio exceeds the set threshold. Otherwise, the detection is regarded as invalid. mAP@50 only evaluates model detection capability based on a single overlap threshold, while mAP@50:95 takes varying overlap degrees into account, enabling a more comprehensive and accurate evaluation of model detection performance. Precision and recall are computed at a confidence threshold of 0.001, while mAP@50 is derived from the area under the precision–recall curve across all confidence thresholds. This setup allows mAP@50 to reflect the model’s overall detection capability across different confidence levels, whereas the reported precision and recall provide a snapshot of performance at a specific low-confidence cutoff.

## 4. Experimental Results and Analysis

### 4.1. Experimental and Analysis

#### 4.1.1. Ablation Experiments Analysis

**Figure 5:**
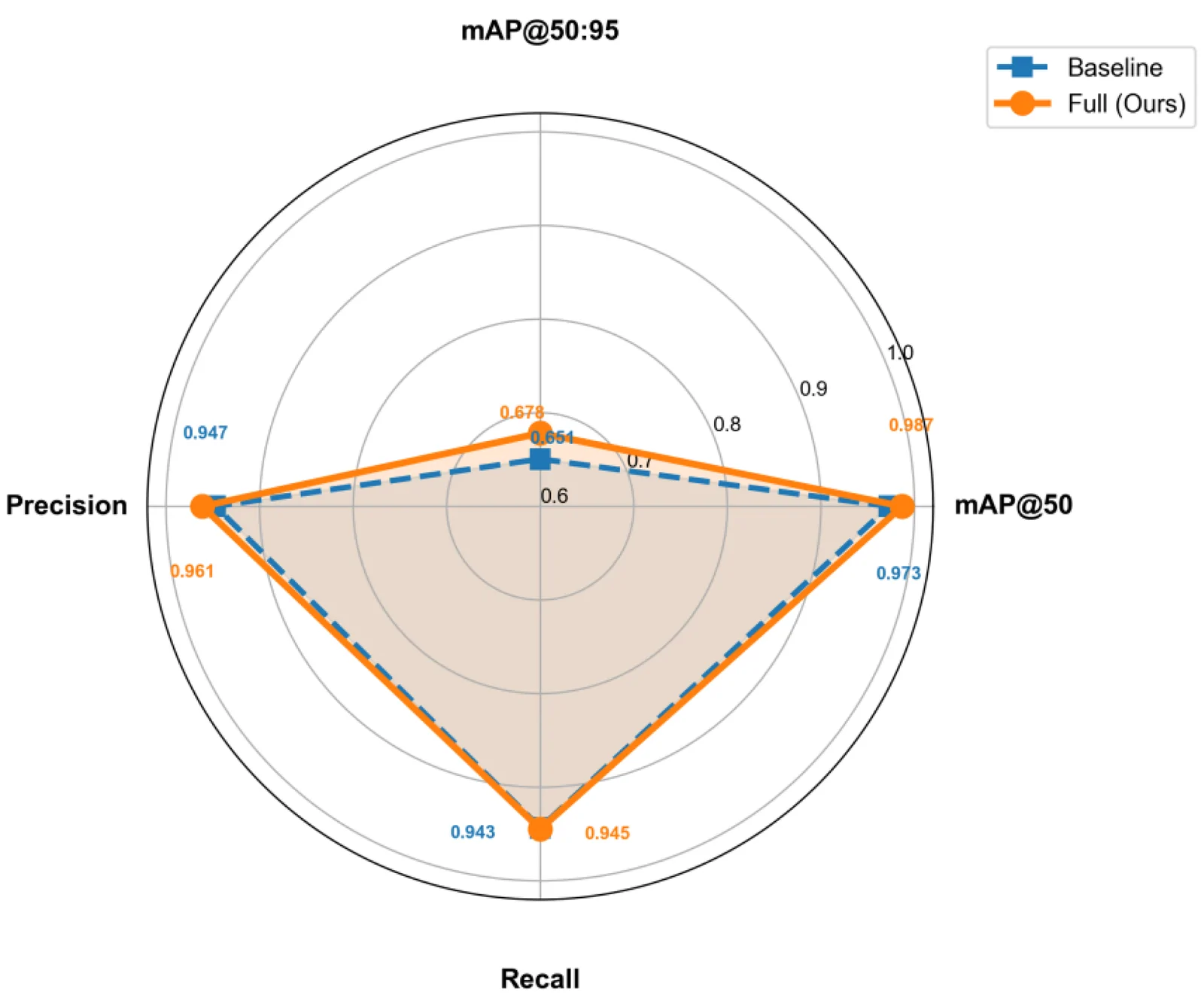
Model performance radar chart (SSDD).

To validate the efficacy of the three improved modules put forward in this work, ablation tests are carried out over the SSDD and HRSID datasets. We gradually add each improved module to the Baseline network and compare the detection accuracy across different experiments. The experimental outcomes derived from the SSDD dataset are summarized in Table 4 (all metrics are evaluated on the test set using the best trained model), and the data comparison of SSDD is shown in Figure 5. 

#### 4.1.2. Statistical Bounds on Training Stochasticity

To quantify the impact of training randomness on the ablation conclusions, we repeat the SSDD Baseline and Full configurations with three independent random seeds {42, 123, 2024} under the identical protocol: 7:2:1 stratified split, 416 × 416 input, AdamW with an initial learning rate of 5 × 10^−4^, batch size of 8, and patience of 50, as summarized in Table 5.

The intermediate ablation configurations (a)–(c) were evaluated with a single seed and are interpreted as indicative trends only.

A paired one-sided *t*-test yields t = 9.18 with df = 2 and *p* = 0.0058 < 0.01. Given the small sample size (*n* = 3), we additionally report the paired effect size (Cohen’s d = 5.30), indicating a systematic improvement. Baseline’s intrinsic 3-seed spread is 0.9640–0.9727 (±0.36 pp), giving a two-sigma interval of [0.9613, 0.9757]. Any single-seed ablation result falling within this band is of the same magnitude as normal training variability, as listed in Table 6.

Configuration (a) achieves an mAP@0.5 of 0.9632. This value falls within the Baseline ±2σ range and is only 0.19 pp higher than the lower bound. This apparent performance drop is explained by normal training variance and lacks statistical significance. The true value of ACmix emerges through synergy with the other modules. When ACmix serves as the attention carrier for NG and is combined with SW-CIoU, the Full model obtains an mAP@0.5 of 0.9870 at the same seed=42, which is 1.85 pp higher than the Baseline mean. The three-seed average of the Full model is 0.9803, which exceeds the Baseline 2σ upper bound (p≈0.0058 p=0.0058 from a paired one-sided *t*-test), confirming genuine effectiveness of the module combination.

Adding NG on top of ACmix (configuration b) on SSDD reduces recall from 0.9432 to 0.9031 (−4.01 pp) and increases precision from 0.9470 to 0.9565 (+0.95 pp). This is a standard precision–recall trade-off caused by conservative noise suppression, rather than genuine performance degradation. The impact of NG varies strongly across datasets. SSDD and HRSID have similar median ENL values (4.00 vs. 4.07), but SSDD exhibits a much larger ENL variance (34.26 vs. 2.43). Consequently, the global correlation between gate activation and the image SNR is weak on SSDD (r≈0,p=0.87). In this regime, NG tends toward conservative suppression and incorrectly discards a small number of low-confidence true ship detections in high-ENL coastal scenes, resulting in the observed −4.01 pp recall drop. The experimental results on the HRSID dataset are summarized in Table 7.

**Figure 6:**
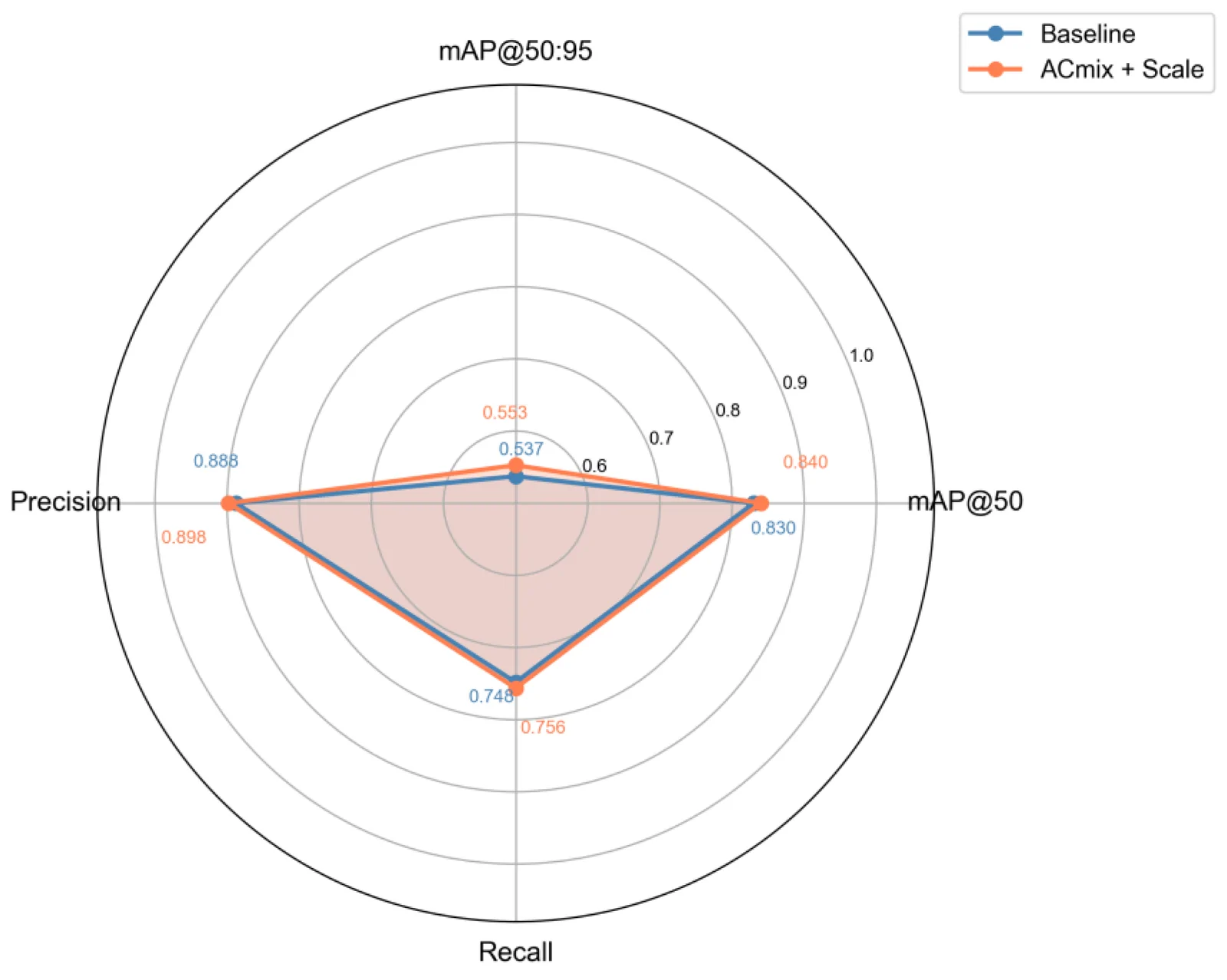
Model performance radar chart (HRSID).

All HRSID results in this table are obtained with Strategy B (two-stage training with the NG frozen in Stage 2), selected as the final HRSID configuration. The SSDD results are obtained with single-stage joint training (Strategy A). All relative changes are computed against the YOLO11 Baseline of the same dataset, and the data comparison of HRSID is shown in Figure 6.

From the ablation results, the standalone ACmix hybrid attention module brings overall performance gains on both the SSDD and HRSID datasets. On the HRSID dataset, precision (P) slightly increases from 0.8880 to 0.8884 (+0.04 pp), and recall (R) rises from 0.7482 to 0.7588 (+1.06 pp). mAP@0.5 increases from 0.8300 to 0.8434 (+1.34 pp), and mAP@0.5:0.95 increases from 0.5374 to 0.5456 (+0.82 pp). On the SSDD dataset, precision (P) increases from 0.9470 to 0.9555 (+0.85 pp). Recall (R) increases from 0.9432 to 0.9435 (+0.03 pp). However, mAP@0.5 decreases by 0.95 pp and mAP@0.5:0.95 by 1.33 pp. These results indicate that the module, by introducing a global receptive field, helps mine more discriminative semantic clues. This method cuts down the total count of missed targets and false positives of marine vessels. Meanwhile, it mitigates the adverse effects caused by background clutter and faint feature information of tiny ships in SAR images to some extent. This “abnormal” degradation stems from two defects in the SSDD dataset. First, the multiplicative speckle noise in SSDD images interferes with the global self-attention mechanism of ACmix, which makes the attention distribution scattered. This weakens the model’s ability to rank feature map confidence scores, leading to a significant decline in localization performance under high IoU thresholds. Second, the regression loss tends to overfit large and easy samples. The gradient update weights are biased toward large targets, which weakens the model’s ability to optimize bounding box constraints for small targets. As a result, the false alarm rate for small targets remains high.

To address the localization degradation caused by multiplicative speckle noise in SAR images, we introduce a channel SAR noise suppression gate before the ACmix module, forming the ACmixSAR module. The gating mechanism uses feature recalibration. It forces the self-attention to focus on strong edge regions with physical scattering significance. This greatly improves the refinement of regression boxes and significantly increases the number of positive samples that meet high IoU thresholds. Experimental results show that the model achieves comprehensive performance improvements after introducing ACmixSAR. On the SSDD dataset, compared with the original ACmix, mAP@0.5 increases to 0.9702 (+0.70 pp), mAP@0.5:0.95 increases to 0.6509 (+1.36 pp), and precision (P) further increases to 0.9565 (+0.10 pp). The improvement in recall indicates that the gate not only suppresses interference but also enhances the responses of effective channels. This activates the features of weak targets that were previously masked by noise. On the HRSID dataset, overall detection performance is also improved, accompanied by a drop in precision (P). This suggests that HRSID is a high-resolution SAR dataset containing abundant fine-grained textures. The noise suppression module may mistakenly suppress these subtle textures as irrelevant clutter, which degrades the feature extraction capability of the network.

To tackle the missed detections of tiny targets caused by large variations in ship scales and scale imbalance in bounding box regression loss within SAR port scenes, we introduce the scale-weighted CIoU loss function. Without modifying the feature extraction branch of ACmix, this loss dynamically adjusts gradient magnitudes according to target sizes and assigns larger loss weights to small ships. The experimental results show that under this configuration, on the SSDD dataset, precision (P) significantly increases from 0.9470 to 0.9667 (+1.97 pp), and mAP@0.5:0.95 increases from 0.6506 to 0.6534 (+0.28 pp). On the HRSID dataset, precision (P) increases from 0.8880 to 0.8979 (+0.99 pp), recall (R) increases from 0.7482 to 0.7562 (+0.80 pp), mAP@0.5 increases from 0.8300 to 0.8397 (+0.97 pp), and mAP@0.5:0.95 increases from 0.5374 to 0.5525 (+1.51 pp). Although the improvement in small target localization accuracy is relatively limited due to the spatial resolution constraints of SAR images, these results are sufficient to verify the effectiveness of the small-scale strategy. The detailed hyperparameter settings of the scale-weighted CIoU loss and other training strategies are summarized in Table 8, while the performance results of three training strategies across two datasets (test set, fixed seed = 42) are listed in Table 9.

We select Strategy B as the final HRSID configuration for three reasons: (i) it achieves the highest test recall (78.02% vs. 76.94% for Strategy C), the priority metric for miss-detection-critical maritime surveillance; (ii) freezing the NG in Stage 2 preserves the noise-suppression behavior learned in Stage 1 and avoids gate drift during detection fine-tuning; (iii) the mAP@0.5 advantage of Strategy C over B (0.8484 vs. 0.8454, 0.30 pp) is within seed-level fluctuation (±0.36 pp), whereas the recall gap (+1.08 pp) favors B. Strategy A (single-stage joint training) is retained for SSDD, where it is significantly better across three seeds (Table 5). All HRSID results in Table 7 and subsequent analyses therefore use Strategy B.

The ablation results above verify that NG and SW-CIoU address feature suppression and regression imbalance, respectively. However, the strategy comparison in Table 9 reveals a striking dataset-dependent behavior when both modules are optimized together. On SSDD, joint training (Strategy A) is already optimal (mAP@0.5=0.9870), because SSDD’s low gate–SNR correlation (r≈0,p=0.87) means NG remains mostly passive and introduces little gradient interference. On HRSID, by contrast, joint training (A) yields the lowest recall (0.7430) and a clear mAP@0.5 loss (−0.84 pp vs. B). The gate–SNR correlation is strong but layer-dependent in sign on HRSID (|r| = 0.42–0.81 across the four gated layers, *p* < 0.001), while on SSDD it is weak and non-significant (global r = −0.01, *p* = 0.87). We report these as evidence of layer-specific association only, without claiming a causal suppression mechanism. Simultaneously, SW-CIoU amplifies gradients on small-vessel residuals that are precisely the ones NG is suppressing. This gradient-level tug-of-war destabilizes Stage-1 learning and causes NG to over-suppress texture-rich coastal features, which directly explains the precision dip observed in configuration (b).

To settle this contradiction, this paper adopts a dataset-adaptive training strategy. For SSDD where NG is largely passive, standard joint training (Strategy A) suffices. For HRSID where NG is highly active, we propose a two-stage decoupling procedure. Stage 1 trains the Noise Gate (and ACmix) under vanilla CIoU until convergence, allowing NG to learn robust speckle suppression statistics without gradient interference from SW-CIoU. Stage 2 then freezes all Noise Gate parameters (requires_grad = False, Strategy B), reduces the initial learning rate to $1\times 10^{-4}$, and enables SW-CIoU for up to 150 epochs. This decoupling eliminates the backpropagation tug-of-war and achieves collaborative gains from both modules. On the SSDD dataset (Strategy A), precision (P) increases from 0.9470 (Baseline) to 0.9612 (+1.42 pp), recall (R) increases from 0.9432 to 0.9446 (+0.14 pp), mAP@0.5 increases from 0.9727 to 0.9870 (+1.43 pp), and mAP@0.5:0.95 significantly increases from 0.6506 to 0.6783 (+2.77 pp). On the HRSID dataset (Strategy B), recall (R) increases from 0.7482 to 0.7802 (+3.20 percentage points, corresponding to a relative growth of 4.28%), mAP@0.5 increases from 0.8300 to 0.8454 (+1.54 pp), and mAP@0.5:0.95 increases from 0.5374 to 0.5539 (+1.65 pp), confirming that the two-stage decoupling strategy effectively resolves the gradient conflict on high-speckle SAR datasets. These patch-level gains are partially attributable to scene overlap between training and test patches, as confirmed by the leakage-free scene-level validation in Section 4.1.4.

#### 4.1.3. Training Convergence and Loss Function Analysis

**Figure 7:**
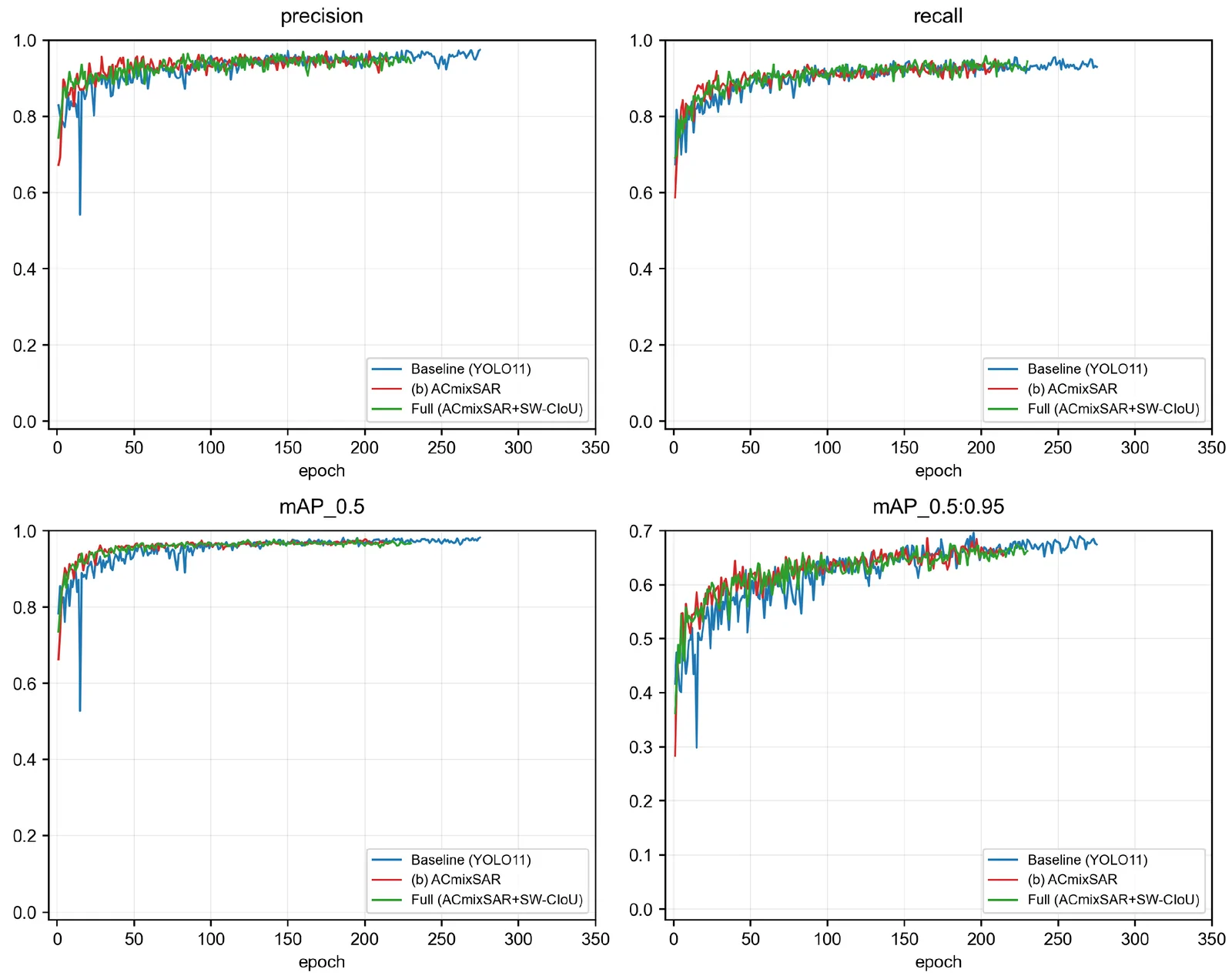
Comparison of performance convergence curves between Baseline and Full models.

To further validate the performance of the proposed modules, Figure 7 plots the convergence curves corresponding to three models trained on the SSDD dataset: Baseline (YOLO11), ACmixSAR (YOLO11-ACmixSAR, configuration (b)), and Full (YOLO11-ACmixSAR+SW-CIoU). All three models are trained under the identical joint-training protocol. As observed in the figure, after introducing the ACmixSAR module (configuration b), the model achieves better performance than Baseline across all metrics, with a clear advantage in mAP@0.5:0.95. The Full model (ACmixSAR+SW-CIoU) further improves all metrics, and mAP@0.5 finally reaches 0.9870. The Noise Gate improves feature quality through channel suppression, and the scale-weighted CIoU loss optimizes bounding box regression for small targets. These two components work together to enhance model performance. Moreover, none of the training curves show obvious overfitting, indicating that the models have good generalization ability.

**Figure 8:**
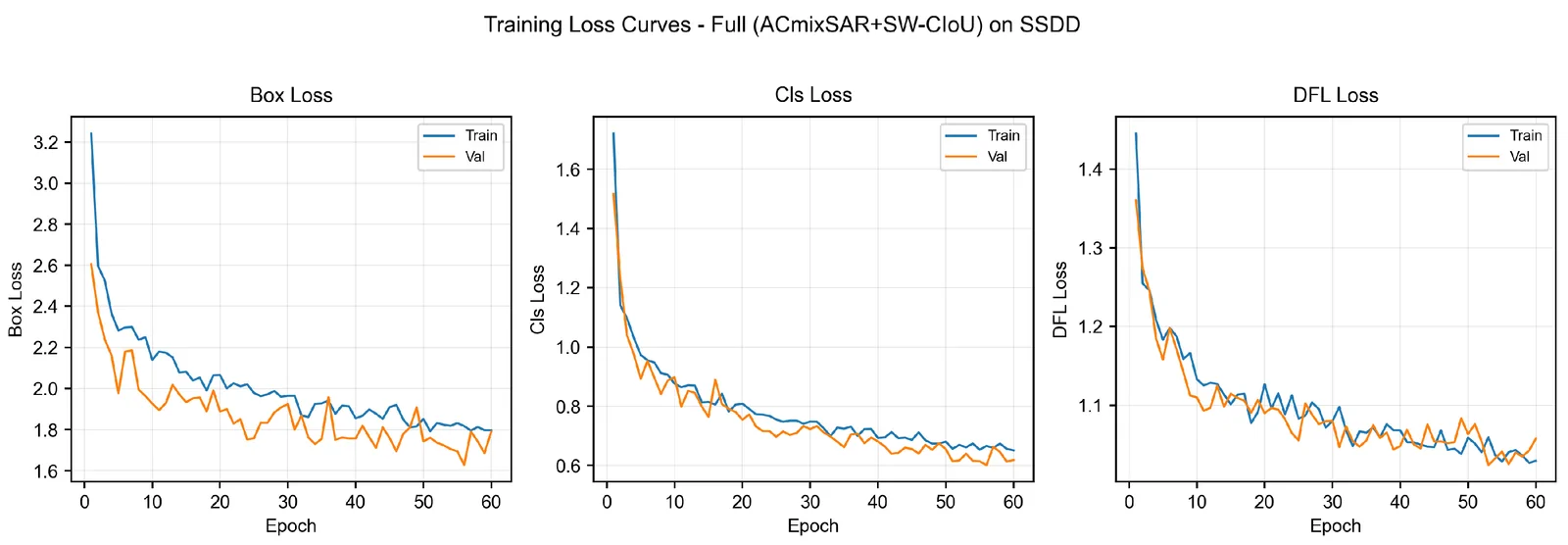
Loss curves of ACmixSAR-YOLO11 during training and validation.

To further verify the stability and generalization ability of the model training process, Figure 8 displays the loss convergence curves for the Full (SSDD) model throughout training. Box Loss, Cls Loss, and DFL Loss stand for bounding box regression loss, classification loss, and distribution focal loss, respectively. As observed in the figure, all three loss curves show a rapid downward trend in the early stage of training, indicating that the model can quickly capture the basic features of ship targets. As the number of iterations increases, both training loss and validation loss decrease smoothly and simultaneously, without showing typical overfitting. After approximately 40 epochs, all loss curves level off, indicating that the model has reached a stable convergence state.

From the results illustrated in Table 4 and Table 7 and Figure 5, Figure 6, Figure 7 and Figure 8, the proposed network achieves optimal detection precision while possessing favorable convergence performance and generalization capacity throughout the training phase. These results lay a solid foundation for the fairness and reliability of the follow-up comparative tests.

#### 4.1.4. Scene-Level Split Validation

To quantify the influence of scene overlap in the original patch-based split of HRSID, we construct a strictly leakage-free scene-level partition where all patches from a single raw SAR scene are entirely assigned to train, validation, or test set without cross-set leakage. The Full model and YOLO11 Baseline are evaluated under identical training hyperparameters. The quantitative results are summarized in Table 10.

On the strictly leakage-free scene-level split, the Full model performs on par with the Baseline in mAP@0.5 (difference −0.3 pp); because this evaluation used a single training seed (42), no seed-level significance claim is made. The ±0.4 pp figure is the seed-level standard deviation observed on SSDD in the three-seed experiment (0.9727/0.9689/0.9640; population σ ≈ 0.36 pp, rounded to 0.4 pp) and is quoted only as an approximate reference. Meanwhile, the Full model retains higher mAP@0.5:0.95 (+0.6 pp) and precision (+1.3 pp). The performance gains observed under the conventional patch-level split therefore partly reflect scene overlap and do not fully transfer to a leakage-free setup. Accordingly, we position HRSID as supplementary experimental evidence, while SSDD (image-level split, leakage-free by design) serves as the primary benchmark for our detection experiments. The scene-level re-evaluation has been completed, and the corresponding quantitative results are summarized in Table 10.

### 4.2. Comparison with State-of-the-Art Methods

To fully assess the performance of the proposed ACMixSAR-YOLO11 for SAR vessel detection tasks, we compare its optimal results on the SSDD dataset with several classic object detection algorithms. The compared algorithms include FCOS, Faster R-CNN, YOLO5s, YOLO8s, and YOLO11s. All competing models are trained with identical dataset partitioning and identical hyperparameter configurations. The quantitative outcomes of this set of experiments are listed in Table 11, and the corresponding comparison results on the HRSID dataset are presented in Table 12.

From the experimental results, as an early anchor-free one-stage model, the traditional algorithm FCOS is limited by speckle noise and blurred ship contours in SAR images. Its precision is only 0.8614, recall is only 0.7654, and mAP@0.5 and mAP@0.5:0.95 are as low as 0.8256 and 0.5185, respectively. Its overall detection capability is much weaker than two-stage algorithms and the lightweight YOLO series. The traditional algorithm Faster R-CNN improves accuracy with its two-stage detection architecture. However, its recall is only 0.9032. It suffers from severe missed detections of small ships and nearshore occluded ships in SAR images. Its mAP@0.5:0.95 is only 0.5978.

Three lightweight YOLO models, YOLO5s, YOLO8s, and YOLO11s, achieve significantly better results than FCOS and Faster R-CNN across all metrics. Among all Baseline models, YOLO5s delivers the highest overall detection accuracy, reaching an mAP@0.5 value of 0.9757. YOLO8s and YOLO11s show a slight decrease in mAP@0.5:0.95 compared to YOLO5s. This is due to the minor accuracy loss caused by lightweight optimization, which is consistent with the iterative trend of the YOLO series. Accordingly, we take YOLO11s as the Baseline. As the newest lightweight YOLO model, it has balanced precision and recall and requires fewer parameters and less computation than YOLO5s and YOLO8s.

In terms of recall, mAP@0.5 and mAP@0.5:0.95, ACmixSAR-YOLO11 achieves superior performance over all compared reference algorithms on both datasets. On SSDD, the proposed model also outperforms all competitors in precision. On HRSID, the model achieves the highest recall at the cost of a slight precision drop compared with the YOLO11 Baseline. Overall, ACmixSAR-YOLO11 is well-suited for SAR ship detection tasks: it reduces missed detections and improves localization accuracy, achieving competitive comprehensive detection performance.

Table 13 supplements the strict head-to-head comparison (Table 11 and Table 12) with a literature-level reference comparison against five recently published SAR-specific detectors on both SSDD and HRSID. Four of the five SARspecific detectors use 640 × 640 input size. CSSYOLO, DBYOLO, LPEDet, SADWDet and MSADET are tested on SSDDv2 and official HRSID with the 7:2:1 train/val/test split.

On SSDD, our method achieves 98.70%, outperforming all five competitors, including CSS-YOLO (98.6%), SADW-Det [32] (98.3%), MSA-DET (98.1%), DB-YOLO [33] (97.8%), and LPEDet [34] (97.4%). Notably, four of the five methods use a 640 × 640 input, which is 53.8% larger than our 416 × 416, and MSA-DET reports results on SSDD v2, a label-augmented version with richer annotations. Despite operating at a lower resolution and on an older dataset version, our method still achieves superior performance, strongly validating the effectiveness of our design.

On HRSID, the reported mAP@0.5 values of published methods range from 89.7% to 94.8%, which are higher than our 84.54%, showing an apparent gap of 5.16 to 10.26 percentage points. However, this discrepancy originates mainly from inconsistent experimental protocols. First, these competing methods adopt 640 × 640 input images. Given that 96.9% of ship targets in HRSID are extremely small, the larger input resolution alone can yield a notable mAP gain. Second, HRSID has no official standard train/val/test split, and different partitioning strategies can introduce substantial mAP fluctuations. More importantly, our HRSID recall reaches 78.02%, the highest among all YOLO-series Baselines, which indicates fewer missed ship detections.

In summary, ACmixSAR-YOLO11 outperforms all compared published SAR-specific detectors on the standard SSDD benchmark, even under conservative protocol handicaps. The performance gap on HRSID is mainly attributed to differences in resolution and data splits rather than architectural shortcomings, and our recall remains the best in practical surveillance scenarios.

### 4.3. Efficiency and Multi-Scale Performance Analysis

To fully evaluate our method, we conduct experiments from two aspects: model efficiency and multi-scale detection capability. Table 14 compares the parameter count, computational complexity, and inference speed of different detectors. Table 15 presents the per-scale AP results under COCO area thresholds.

Parameter and FLOPs counts are identical for both datasets because the two Baselines share the same network configuration. Inference speed was measured under a single protocol (one RTX 5070, batch size 1, imgsz 416, 30 warm-up and 200 measured iterations, averaged over three repeated runs).

From Table 14, we compare the model complexity and inference speed of all methods on both SSDD and HRSID datasets. On SSDD, the Baseline model contains 9.69M parameters and achieves 156.1 FPS with 21.5 GFLOPs. After introducing ACmix, the parameter count increases to 11.70M (+20.7%) and the computational cost to 25.4 GFLOPs (+18.1%), while FPS drops to 123.2. Adding the NG module brings the parameters to 11.75M at the same 25.4 GFLOPs, with FPS recovering to 131.8. On HRSID, the Baseline model achieves 156.1 FPS with 21.5 GFLOPs. The Full model reaches 117.9 FPS with 11.75M parameters and 25.4 GFLOPs, maintaining comparable efficiency.

In terms of real-time performance, our Full model reaches 131.8 FPS on SSDD and 117.9 FPS on HRSID, both far exceeding the real-time detection threshold (≥30 FPS). Compared with FCOS (73.0 FPS on SSDD/77.1 FPS on HRSID) and Faster R-CNN (67.8 FPS on SSDD/69.0 FPS on HRSID), our method achieves 80.5%/52.9% speed improvements over FCOS and 94.4%/70.9% over Faster R-CNN on SSDD/HRSID, respectively, while reducing parameters by 63.4% and 71.6%. When compared with YOLO5s (212.0/186.5 FPS) and YOLO8s (206.0/209.9 FPS), our model maintains comparable parameter size (11.75 M) and computational cost (25.4 GFLOPs) yet delivers better multi-scale accuracy, as shown in Table 15. These results confirm that our method is both lightweight and practical for real-world deployment.

From Table 15, the Full model exhibits a scale-dependent trade-off rather than uniform improvements. On SSDD, where per-scale APs are near saturation, the Full model’s AP for small ships is 95.71%, marginally below the Baseline (96.31%, −0.60 pp); medium-ship AP changes only marginally (99.17% vs. 99.32%, −0.15 pp), and large-ship AP decreases from 100.00% to 93.19% on this extremely small subset (n = 8), where both models retain 100% recall@0.25 (Table 16), so the gap reflects localization ranking rather than lost coverage. On HRSID, small-ship AP decreases by 2.26 pp relative to the Baseline, medium-ship AP decreases by 0.53 pp, and large-ship AP improves by +12.89 pp, indicating that SW-CIoU primarily rebalances localization quality toward large ships on this dataset. We therefore do not claim a universal improvement in small-ship AP. A complementary recall-oriented analysis (recall@0.25 and miss-detection rate (MR), IoU = 0.25; Table 16) shows that small-ship detection coverage is maintained on both datasets, and the miss rate of the 14 large HRSID instances drops from 7.1% to 0%. Configuration (c) yields an AP of 72.69% for small ships on HRSID (Table 15).

In summary, the results from Table 14, Table 15 and Table 16 together show that our method achieves a good balance between efficiency and multi-scale detection accuracy. The Full model not only maintains real-time inference speed with a moderate parameter increase over the Baseline but also delivers stable and competitive detection coverage for small and medium object scales on both SSDD and HRSID, demonstrating its practical value for SAR ship detection.

### 4.4. Visualization of Detection Results

**Figure 9:**
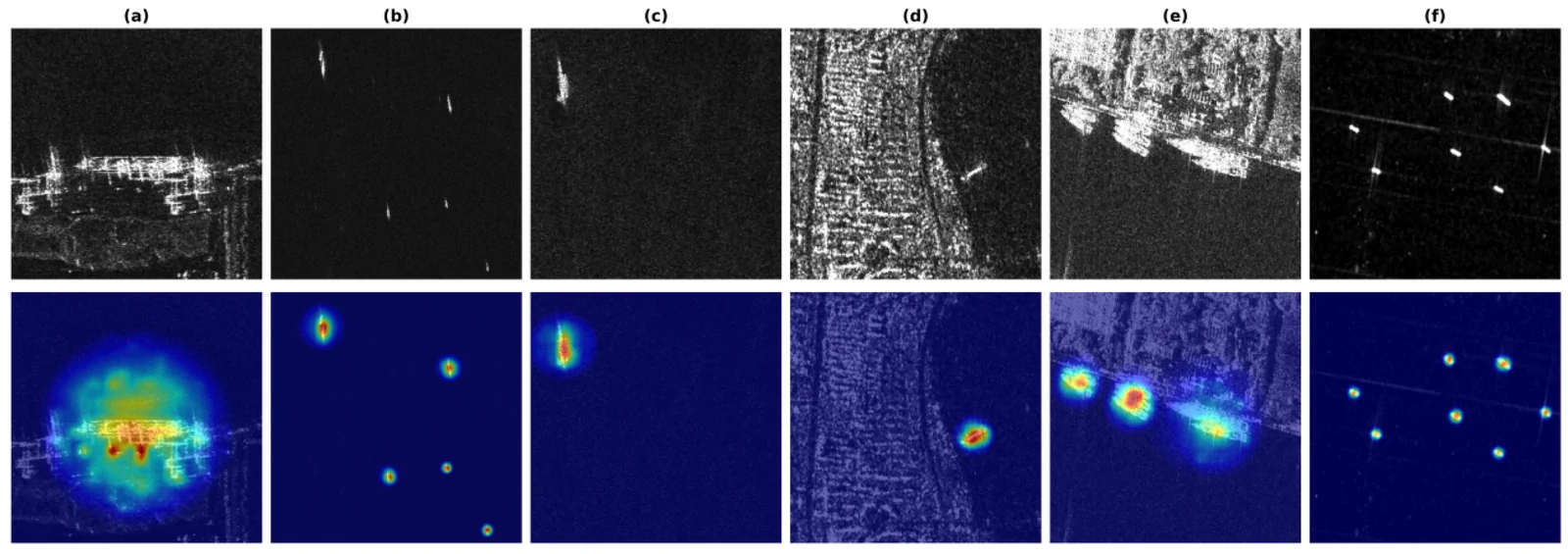
Grad-CAM heatmaps of neck feature fusion layer in ACmixSAR-YOLO11. (**a**) SAR image and Grad-CAM heatmap for ship scene 1; (**b**) SAR image and Grad-CAM heatmap for ship scene 2; (**c**) SAR image and Grad-CAM heatmap for ship scene 3; (**d**) SAR image and Grad-CAM heatmap for ship scene 4; (**e**) SAR image and Grad-CAM heatmap for ship scene 5; (**f**) SAR image and Grad-CAM heatmap for ship scene 6.

**Figure 10:**
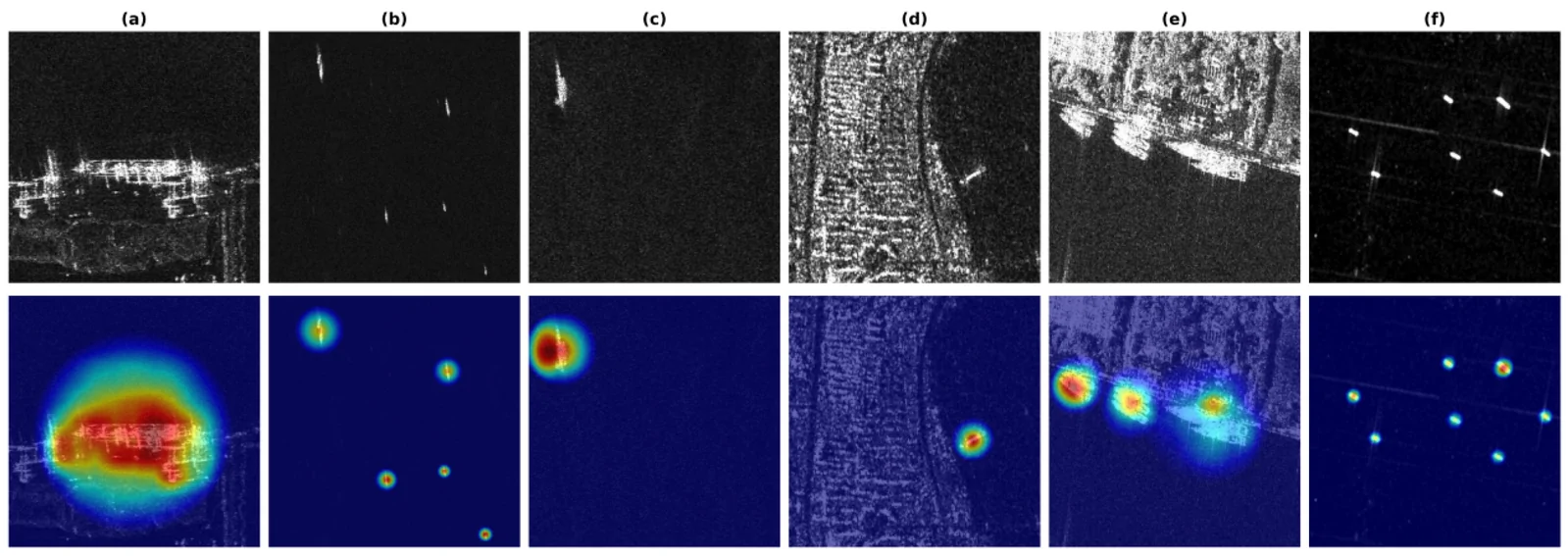
Grad-CAM heatmap of the detection head front layer in ACmixSAR-YOLO11. (**a**) SAR image and Grad-CAM heatmap for ship scene 1; (**b**) SAR image and Grad-CAM heatmap for ship scene 2; (**c**) SAR image and Grad-CAM heatmap for ship scene 3; (**d**) SAR image and Grad-CAM heatmap for ship scene 4; (**e**) SAR image and Grad-CAM heatmap for ship scene 5; (**f**) SAR image and Grad-CAM heatmap for ship scene 6.

This paper employs Grad-CAM feature visualization. The C3k2_ACmixSAR block in the neck and the front layer of the detection head are selected as observation nodes. Figure 9 shows the Grad-CAM heatmap of the C3k2_ACmixSAR block in the neck, and Figure 10 shows the Grad-CAM heatmap from the first layer of the prediction head. By comparing these two sets of visualization results, we can observe that the neck heatmap has a smaller activation area and focuses on local edge and texture features of ships. The detection head heatmap has a larger activation area and focuses on global contours and positional information of ship targets. Despite their differences in granularity, the red core peaks in both heatmaps are strictly confined to the ship targets. There is no redundant background activation. This verifies that the ACmixSAR module can effectively focus on targets and suppress clutter at all stages of feature extraction, with features gradually improving from local to global, as seen from the neck to the detection head heatmap. It also provides a feature-level explanation for the model’s high detection accuracy.

**Figure 11:**
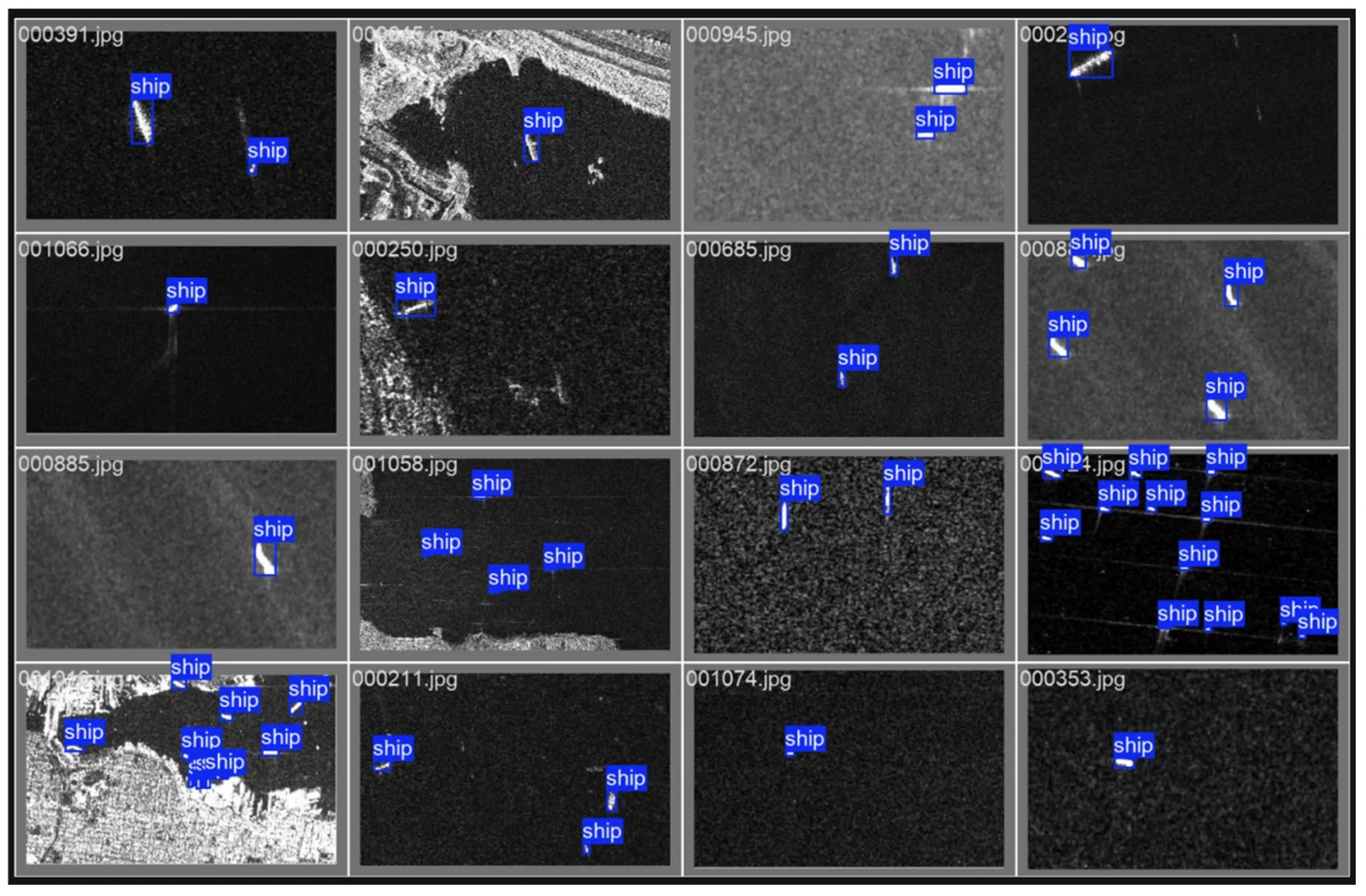
Detection visualization of ACmixSAR-YOLO11.

To intuitively display the detection capability of our designed network, we visualize prediction outputs generated by ACmixSAR-YOLO11 on several sample images from the SSDD dataset in Figure 11. The blue bounding boxes within the subfigures stand for the predicted targets yielded by our algorithm. It can be clearly seen that this network precisely locates marine vessels under diverse complex scenarios, such as isolated vessels on the open ocean, crowded ship clusters inside harbors, and tiny coastal vessels close to shorelines. These visualized cases demonstrate the robust detection capability of the presented framework.

## 5. Conclusions

To solve the problems of large scale changes, strong speckle noise, and sea clutter in SAR ship detection, this paper proposes a lightweight model called ACmixSAR-YOLO11 based on YOLO11. The model adds ACmix hybrid attention to the neck network and uses learnable weights to balance global and local features. It also adds a SARNoiseGate module to reduce low-SNR noise and boost ship features. A small-scale CIoU loss is designed to give higher regression weights to small targets, which balances gradient updates across ships of different sizes. A two-stage training strategy is used to optimize the Noise Gate and scale loss in separate steps, avoiding gradient interference.

Experiments on SSDD, our primary leakage-free benchmark, demonstrate that the combination of the proposed SARNoiseGate and SW-CIoU loss yields consistent performance gains over the YOLO11 baseline. Ablation experiments verify the individual and synergistic contributions of the ACmixSAR module and scale-weighted loss function. Results on HRSID serve as supplementary evidence. The original patch-level split of HRSID introduces scene overlap between training and test samples. When evaluated on a leakage-free scene-level split, the mAP@0.5 improvement disappears, while gains in mAP@0.5:0.95 and precision persist. On HRSID, the SARNoiseGate may suppress subtle fine textures of high-resolution SAR images, leading to a slight precision drop. Overall, this work presents a lightweight real-time detection framework suitable for airborne SAR maritime surveillance and vessel tracking.

Limitations of this work include the dataset-dependent behavior of the SARNoiseGate and the sensitivity of HRSID performance to data splitting strategies. Future work will develop noise suppression modules adaptive to varying SAR image resolutions, design more robust multi-scale loss functions, and explore model slimming strategies to further balance detection accuracy and inference efficiency.

## Figures and Tables

**Table 1:** Statistical validation of SAR speckle coupling.

Analysis Item	SSDD	HRSID
Best fit distribution	Gamma (KL≈0.012)	Gamma (p<0.001)
Gate–SNR correlation	|r| ≤ 0.30, n.s.	|r| = 0.42–0.81, *p* < 0.001
ENL (median)	4.00	4.07
ENL (variance)	34.26	2.43

SSDD—global Pearson r = −0.01 (*p* = 0.87, n.s.), per-layer |r| up to 0.30; HRSID—per-layer |r| = 0.42–0.81 (all *p* < 0.001), global r = −0.10.

**Table 2:** Sensitivity analysis of SW-CIoU scale weights on SSDD dataset.

Config	$w_{s}$ (Small)	$w_{m}$ (Medium)	$w_{l}$ (Large)	mAP@0.5
W0	1.0	1.0	1.0	0.9654
W1	1.2	1.1	1.0	0.9762
W2	1.5	1.2	1.0	0.9804
W3	1.7	1.3	1.0	0.9778
W4	2.0	1.5	1.0	0.9731
T0	1.2	1.0	1.0	0.9715
T1	1.5	1.0	1.0	0.9768

**Table 3:** Dataset specifications.

Parameter	Details	
Dataset name	SSDD	HRSID
Resolution	1~15 m	0.5~3 m
Image size (used)	217 × 214~526 × 646	800 × 800
Total images (used)	1160	3642 (from 5604 raw images)
Ship labels	2456	16,951
Train/val/test	812/232/116	2549/728/365
Small ships (area ratio < 1%)	79.6%	96.9%

**Table 4:** Impact of different improvement sections on detection performance on SSDD dataset.

Algorithm	ACmix	NG	SW-CIoU	P	R	mAP@ 0.5	mAP@ 0.5:0.95
Baseline				0.9470	0.9432	0.9727	0.6506
(a)	√			0.9555	0.9435	0.9632	0.6373
(b)	√	√		0.9565	0.9031	0.9702	0.6509
(c)	√		√	0.9667	0.9400	0.9707	0.6534
Full	√	√	√	0.9612	0.9446	0.9870	0.6783

**Table 5:** Multi-seed replication of SSDD Baseline vs. Full (test set, mAP@0.5).

Model	Seed = 42	Seed = 123	Seed = 2024	Mean ± Std (%)	Δ vs. BL Mean
Baseline	0.9727	0.9689	0.9640	0.9685 ± 0.36 pp	—
Full	0.9870	0.9795	0.9744	0.9803 ± 0.52 pp	+1.18 pp

**Table 6:** Single-seed ablation points vs. Baseline ±2σ stochastic band (SSDD, seed = 42).

Model (Seed = 42)	mAP@0.5	Δ vs. BL Mean	BL ± 2σ [0.9613, 0.9757]
Baseline	0.9727	+0.42 pp	√
(a)	0.9632	−0.53 pp	√
(b)	0.9702	+0.17 pp	√
(c)	0.9707	+0.22 pp	√
Full	0.9870	+1.85 pp	×

**Table 7:** Impact of various improvement parts on detection performance over HRSID dataset.

Algorithm	ACmix	NG	SW-CIoU	P	R	mAP@0.5	mAP@0.5:0.95
Baseline				0.8880	0.7482	0.8300	0.5374
(a)	√			0.8884	0.7588	0.8434	0.5456
(b)	√	√		0.8838	0.7711	0.8364	0.5425
(c)	√		√	0.8979	0.7562	0.8397	0.5525
Full	√	√	√	0.8781	0.7802	0.8454	0.5539

**Table 8:** Hyperparameter configuration comparison.

Hyperparameter	Stage 1 (NG Learn)	Stage 2 (SW-CIoU)	Joint Training
Learning rate (lr0)	0.0005	0.0001 (5× lower)	0.0005
LR schedule (lrf)	0.01 (cosine)	0.001	0.01
Optimizer	AdamW	AdamW	AdamW
Epochs/patience	500/50	150/50	500/50
Batch/imgsz	8/416	8/416	8/416
SW-CIoU	×	√	√ (from start)
Noise Gate frozen	×	√ (Strategy B)/ × (Strategy C)	×
AMP/seed	False/42	False/42	False/42

**Table 9:** Three training strategies × two datasets (test set, strict seed = 42).

	Strategy	P	R	mAP@0.5	mAP@0.5:0.95
SSDD	A. Joint Training	0.9612	0.9446	0.9870	0.6783
	B. Two-Stage (freeze gate)	0.9391	0.9413	0.9730	0.6502
	C. Two-Stage (no freeze)	0.9482	0.9384	0.9640	0.6373
HRSID	A. Joint Training	0.9046	0.7430	0.8370	0.5478
	B. Two-Stage (freeze gate)	0.8781	0.7802	0.8454	0.5539
	C. Two-Stage (no freeze)	0.8961	0.7694	0.8484	0.5547

**Table 10:** Performance comparison on leakage-free scene-level split of HRSID (single seed (42)).

Model	P	R	mAP@0.5	mAP@0.5:0.95
Baseline YOLO11	0.894	0.841	0.904	0.590
Full	0.907	0.827	0.901	0.596
Delta	+1.3 pp	−1.4 pp	−0.3 pp	+0.6 pp

**Table 11:** Comparison of ACmixSAR-YOLO11 model with other models on SSDD dataset.

Algorithm	P	R	mAP@0.5	mAP@0.5:0.95
FCOS	0.8614	0.7654	0.8256	0.5185
Faster R-CNN	0.9362	0.9032	0.8957	0.5978
YOLO5s	0.9494	0.9348	0.9757	0.6659
YOLO8s	0.9377	0.9443	0.9735	0.6539
YOLO11s (Baseline)	0.9470	0.9432	0.9727	0.6506
ACmixSAR-YOLO11	0.9612	0.9446	0.9870	0.6783

**Table 12:** Comparison of ACmixSAR-YOLO11 model with other models on HRSID dataset.

Algorithm	P	R	mAP@0.5	mAP@0.5:0.95
FCOS	0.8584	0.5977	0.6614	0.4658
Faster R-CNN	0.8912	0.6919	0.7418	0.5025
YOLO5s	0.8885	0.7438	0.8290	0.5319
YOLO8s	0.9072	0.7465	0.8312	0.5508
YOLO11s (Baseline)	0.8880	0.7482	0.8300	0.5374
ACmixSAR-YOLO11	0.8781	0.7802	0.8454	0.5539

**Table 13:** Comparison with published SAR-specific ship detectors.

Detector	SSDD	mAP@0.5 vs. Ours	HRSID	mAP@0.5 vs. Ours
Ours	98.7%	—	84.54%	—
CSS-YOLO	98.6%	+0.10	94.8%	−10.26
DB-YOLO	97.8%	+0.90	94.4%	−9.86
LPEDet	97.4%	+1.30	89.7%	−5.16
SADW-Det	98.3%	+0.40	93.7%	−9.16
MSA-DET	98.1%	+0.60	94.2%	−9.66

**Table 14:** Efficiency comparison of different detectors.

Dataset	Algorithm	Params (M)	GFLOPs	FPS
SSDD	Baseline	9.69	21.5	156.1
	(a)	11.70	25.4	123.2
	(b)	11.75	25.4	112.4
	(c)	11.70	25.4	127.5
	Full (Ours)	11.75	25.4	131.8
	YOLO5s	9.11	23.8	212.0
	YOLO8s	11.13	28.4	206.0
	FCOS	32.12	67.9	73.0
	Faster R-CNN	41.35	92.7	67.8
HRSID	Baseline	9.69	21.5	156.1
	(a)	11.70	25.4	118.3
	(b)	11.75	25.4	115.0
	(c)	11.70	25.4	131.0
	Full (Ours)	11.75	25.4	117.9
	YOLO5s	9.11	23.8	186.5
	YOLO8s	11.13	28.4	209.9
	FCOS	32.12	67.92	77.1
	Faster R-CNN	41.35	92.72	69.0

**Table 15:** Multi-scale detection performance of different detectors.

Dataset	Algorithm	AP_Small (%)	AP_Medium (%)	AP_Large (%)
SSDD	Baseline	96.31	99.32	100.00
	(a)	96.23	97.72	82.18
	(b)	96.26	97.95	91.36
	(c)	97.16	99.78	87.13
	Full (Ours)	95.71	99.17	93.19
	YOLO5s	96.91	97.21	95.05
	YOLO8s	95.81	99.57	86.77
	FCOS	84.19	92.07	90.92
	Faster R-CNN	94.09	96.85	98.57
HRSID	Baseline	76.46	97.17	58.73
	(a)	72.21	96.22	70.39
	(b)	72.30	96.11	57.54
	(c)	72.69	96.73	52.91
	Full	74.20	96.64	71.62
	YOLO5s	71.71	96.60	58.64
	YOLO8s	71.71	97.01	57.47
	FCOS	47.01	93.96	71.95
	Faster R-CNN	61.02	96.15	80.48

**Table 16:** Scale-specific recall@0.25 and miss-detection rate on the patch-level split.

Dataset	Scale	n_Ground Truth	R@0.25 Baseline	R@0.25 Full	MR Baseline	MR Full
SSDD	small	241	0.992	0.992	0.8%	0.8%
	medium	92	1.000	1.000	0.0%	0.0%
	large	8	1.000	1.000	0.0%	0.0%
HRSID	small	663	0.961	0.962	3.9%	3.8%
	medium	459	0.996	0.993	0.4%	0.7%
	large	14	0.929	1.000	7.1%	0.0%

## Data Availability

The original contributions presented in this study are included in the article. Further inquiries can be directed to the corresponding author.
